# A case report of jejunum transplantation in the treatment of severe cervical esophageal stricture in patients with dystrophic epidermolysis bullosa

**DOI:** 10.3389/fonc.2023.1157563

**Published:** 2023-11-06

**Authors:** Zhen Xu, Yong Zhang, Yanjiao Hu, Xia Xiu, Bowen Yang, Tianqiao Huang, Yichuan Huang

**Affiliations:** ^1^ Deparment of Otolaryngology Head and Neck Surgery, The Affiliated Hospital of Qingdao University, Qingdao, China; ^2^ Department of Stomatology, The Huangdao District Central Hospital, Qingdao, China; ^3^ Deparment of Pathology, The Affiliated Hospital of Qingdao University, Qingdao, China

**Keywords:** epidermolysis bullosa, esophageal stenosis, dysphagia, jejunum transplantation, surgical treatment

## Abstract

Epidermolysis bullosa (EB) is a rare disorder caused by autosomal genetic variation. Its main clinical features include skin and mucous membrane blisters, erosion, repeated ulcers and scar formation. The lesions mostly involve the skin, oral cavity, digestive system and urinary system. Epidermolysis bullosa complicated with esophageal stenosis is a common gastrointestinal manifestation of this disorder. Currently, there is no cure for EB, and thus symptomatic treatment is usually applied. Here we describe the case of a patient with recessive dystrophic EB complicated with severe esophageal stenosis. The narrow segment of esophagus was removed and the free part of jejunum was transplanted into the esophageal defect to reconstruct the esophagus and restore the patient’s normal swallowing. For patients with EB complicated with severe esophageal stenosis, surgical resection of the diseased esophagus and jejunal transplantation can be used to repair the esophageal and restore normal swallowing pathway, providing an effective treatment for this condition.

## 1 Introduction

Epidermolysis bullosa (EB) is a rare condition caused by genetic variation at autosomal loci. It is mainly characterized by pathological changes such as skin brittleness increase and other structural abnormalities. The clinical features including skin and mucous membrane blisters, erosion, repeated ulcers, scar formation and ultimately carcinoma (1), which mostly involve the skin, oral cavity, digestive system and urinary system. EB can be divided into four categories according to clinical manifestations and pathological features of the skin layer where the blister occurs: epidermolysis bullosa simplex (EBS) with blisters confined to the epidermal layer, junctional epidermolysis bullosa (JEB) with blisters located in the transparent layer of the basement membrane, dystrophic epidermolysis bullosa (DEB) with blisters located below the compact plate, and Kindler epidermolysisi bullosa (KEB) with mixed cutaneous division pattern (2). DEB can be divided into two subtypes: dominant dystrophic epidermolysis bullosa (DDEB), which presents as localized or intermediate DDEB, and recessive dystrophic epidermolysis bullosa (RDEB) with intermediate or severe symptomatology. The clinical symptoms of RDEB are often more severe than those of DDEB and esophageal stenosis is more likely to occur (3).

Esophageal stenosis is more common in congenital developmental abnormalities (4), after surgery and radiotherapy for malignant tumors, chemical erosive injury, and gastroesophageal reflux and eosinophilic esophagitis (5), but the esophageal stenosis caused by genetic variation of the dermatology is rare. For esophageal stricture in patients with dystrophic epidermolysis bullosa, endoscopic esophageal dilation is often adopted to relieve clinical symptoms. Esophageal dilation, to some extent, will aggravate the damage to the esophageal mucosa, leading to re-stenosis. Pope E et al. found that the location of esophageal stenosis, the length and quantity of stenosis, and the use of glucocorticoid were related risk factors for esophageal restenosis. Patients with upper and middle esophageal stenosis, multi-segmental stenosis, and long segmental stenosis without corticosteroids were more likely to have re-stenosis. The traditional EB type did not increase the risk of esophageal restenosis (6). For patients with severe esophageal stenosis, gastrostomy surgery is often used to ensure nutritional supply (7), but it greatly reduces the quality of life of patients. Here we describe a patient with EB, complicated with severe esophageal stricture was successfully treated by surgical resection of the narrow esophagus, repair of the esophagus by free jejunum, and reconstruction of the upper digestive tract. So far, there has been no literature report on the treatment of EB patients with severe esophageal stenosis by this surgical treatment method.

## 2 Case report

**Figure 1:**
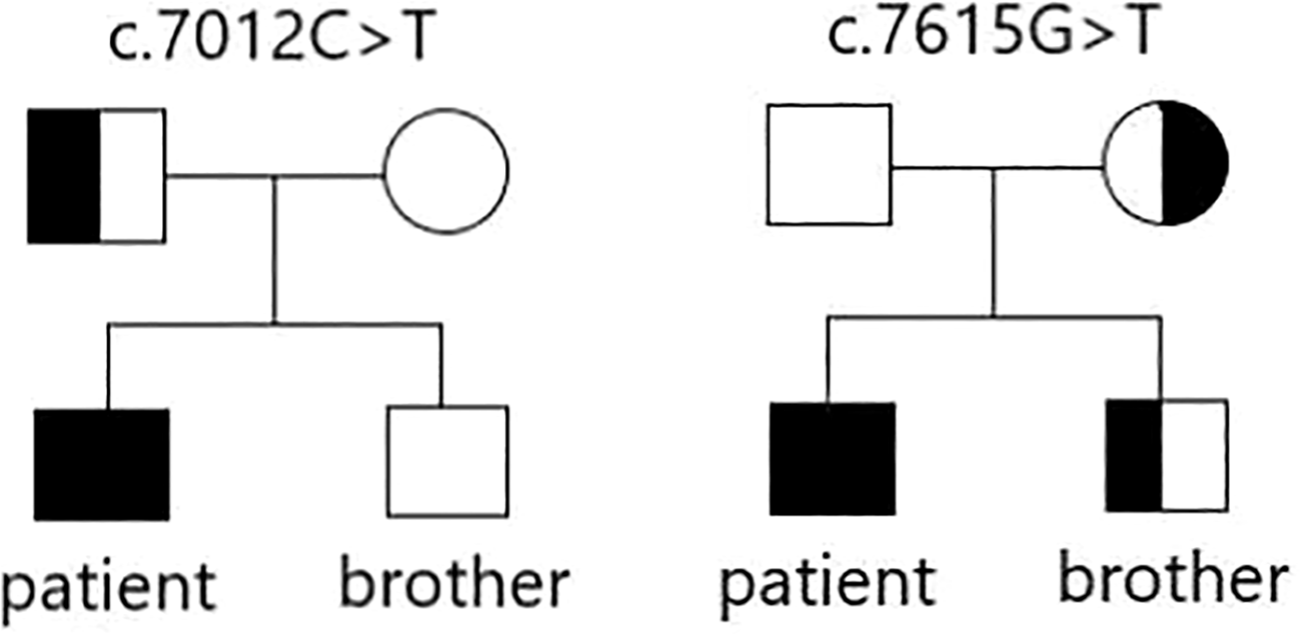
Genetic map of familial disease genes.

A 26-year-old male, after swallowing hard food, developed irregular and intermittent hemoptysis, followed by a sense of swallowing obstruction, which progressively developed into aggravated dysphagia within 2 years. At the age of 28 years, the patient could not swallow a liquid diet normally, and was admitted to the Department of Otolaryngology Head and Neck Surgery, the Affiliated Hospital of Qingdao University in July 2019. Condition on admission: Thin body shape, height: 170 cm, weight: 53 Kg, BMI: 18.3 kg/m^2^. The patient was diagnosed with COL7A1:DEB, Bart type nonspecific epidermolysis bullosa due to “dysphagia with hemoptysis” (data from Beijing Kangso Medical Inspection). A heterozygous variation associated with the rare autosomal recessive dystrophic epidermolysis bullosa (RDEB) type was detected in c.7012C>T and c.7615G>T gene loci of *COL7A1* (type VII collagen gene) located at chr3:48606879. The family disease genetic map is shown in **Figure 1**. In order to alleviate difficulty swallowing, the patient was treated in several hospitals in China and endoscopic esophageal dilation or esophageal stent implantation were utilized without success.

**Figure 2:**
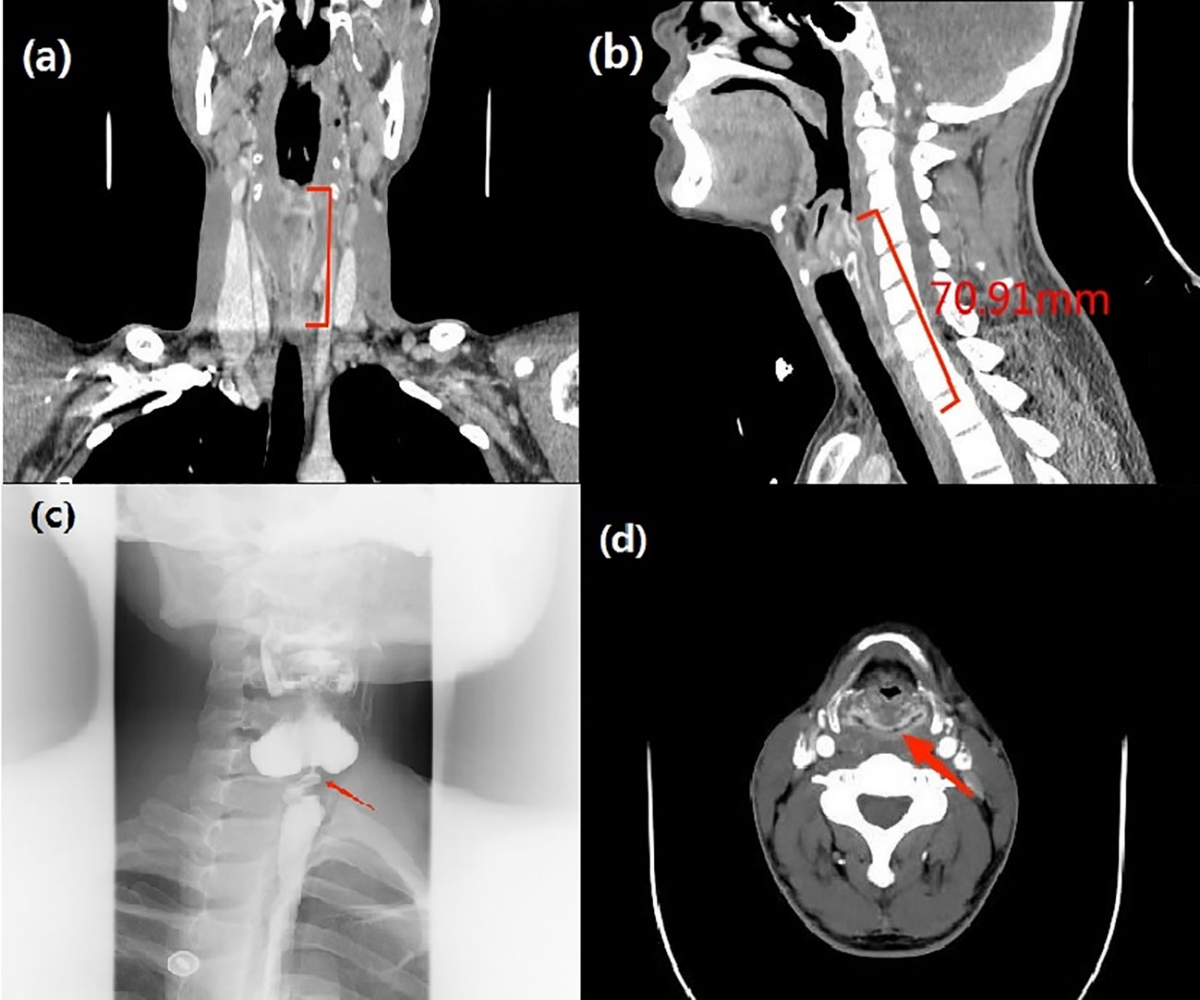
**(A, B)** Neck-enhanced CT: (sagittal and coronal): The mucosa of the upper esophagus was thickened and enhanced to a length of 71 mm. **(C)** Esophageal barium swallowing test: severe stenosis of the horizontal lumen of the C6-C7 vertebral body in the upper esophagus. **(D)** Neck-enhanced CT: (axial): The mucosa of the posterior area of the cricoarytenoid joint showed annular enhancement.

After admission, physical examination, neck CT with contrast (**Figures 2A, B, D**), electronic fiber laryngoscopy, esophageal barium swallowing test (**Figure 2C**) and other examinations were performed. During gastroscopy, a very fine gastroscope could not be passed through the narrow esophagus. At the same time, the patient received intravenous nutritional support. We also organized consultation and discussion of difficult cases from multiple disciplines such as otolaryngology head and neck surgery, microsurgery, gastrointestinal surgery, nutrition, dermatology, etc.

After adequate preoperative preparation, we performed a resection of the narrow esophagus, free jejunum transplantation, and pharyngeal cavity and esophageal anastomosis. About 6 cm long transverse incision was made at the cricoid cartilage level. Subsequently, the superior thyroid arteries and veins were isolated and protected, the right gland lobe of the thyroid was turned up to fully protect the recurrent laryngeal nerve and parathyroid gland, and the tracheoesophageal furrow was exposed to evaluate the narrow esophagus. Obvious stenosis and scarring were observed from the entrance of the esophagus to the subosternal fossa. The otolaryngology head and neck surgeon removed the narrow cervical esophagus, and the defect length was about 8 cm. The gastrointestinal surgeon selected the jejunum about 100 cm from the Qu’s ligament and separated the jejunum about 12 cm in length. The free intestinal section was moved to the upper and lower end of the esophageal defect for annular anastomosis, and a No. 10 gastric tube was inserted into the stomach. The microsurgeon anastomosed one mesenteric artery from the free bowel to the right superior thyroid artery and one vein to the internal jugular vein to ensure normal blood supply to the free jejunum.

**Figure 3:**
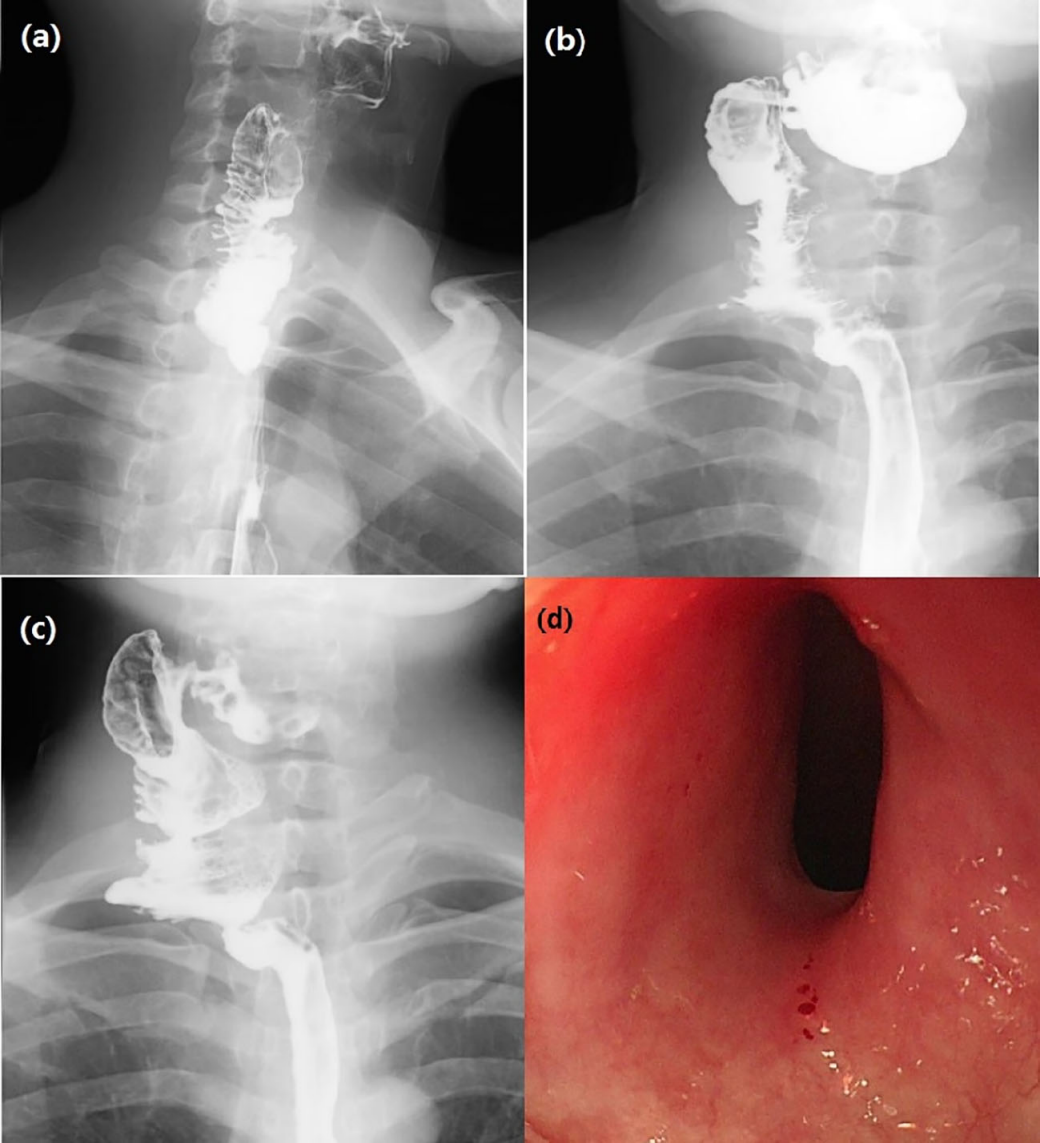
**(A)** Esophagus swallowing iodine oil test on day 18 after surgery showed unobstructed anastomosis without leakage of contrast agent. **(B)** Barium swallow test 5 months after surgery: The contrast agent emptying time of larynx was delayed, and the esophageal lumen was good. **(C)** Esophago swallowing test 29 months after surgery: intestinal tubular shadow could be seen at the upper end of the esophagus, and the upper anastomosis was slightly narrow. **(D)** Gastroscopy 31 months after surgery: The upper edge of the anastomosis was 16 cm from the incisor, the mucosa was smooth, the lumen was slightly narrow, and the body of the ordinary gastroscope passed normally. The lower edge of the anastomosis was spacious. The mucosa of the esophageal lumen was smooth and without ulceration.

After surgery, broad spectrum antibiotics were given to prevent infection. In addition, low molecular weight heparin sodium 41 million IU Q8h anticoagulant was administered for 1 week and papaverine hydrochloride 30 mg bid intramuscular injection to prevent blood vessel spasm, supplemented by continuous gastrointestinal decompression, omeprazole sodium 40mg IV QD inhibited gastric acid secretion. Traditional Chinese acupuncture combined with umbilical moxibustion was used to promote the recovery of intestinal function by daily acupuncture at Tianshu point, Daheng Point, Qihai point and other acupoints. On day 1-3 after surgery, bedside ultrasound doppler was used to assess blood flow in the transplanted jejunal anastomotic vessels, and demonstrated normal blood flow. Hematoma in the operative area of the neck appeared on day 3 after surgery, which was considered a side effect of anticoagulant drugs. Since the patient had no dyspnea, anticoagulant drugs were not stopped, but appropriate pressure drainage was administered on the operative cavity. On day 7 after surgery, local pain was reduced, intestinal function recovered, and no signs of local infection were observed. The patient was given a nasal feeding Enteral Nutritional Suspension (TPF) and after about 5 hours, abdominal pain, diarrhea and bloody stool appeared. Suspension with replacement TPF was replaced by Enteral Nutritional Suspension (SP) and Montmorillonite Power 3g TID was used to control diarrhea. Lactobacillus and Enterococcus Capsules were used to regulate intestinal flora. Sodium Chloride Physiological Solution 50 ml and Octreotide Acetate 0.3 mg was administered intravenously at a rate of 4 ml/h to control intestinal secretion. Noradrenaline Bitartrate was diluted with saline to a ration of 1:25 and injected with a nasal feeding pump to relieve the symptom of intestinal bleeding. Bloody stool lasted for about 8 days after surgery. On day 15 after surgery, bloody stool disappeared and the neck and abdominal drainage tubes were removed. On day 18 after surgery, iodine oil swallowing test (**Figure 3A**) revealed no obvious leakage of contrast agent. The patient began to swallow a liquid diet without difficulty.

**Figure 4:**
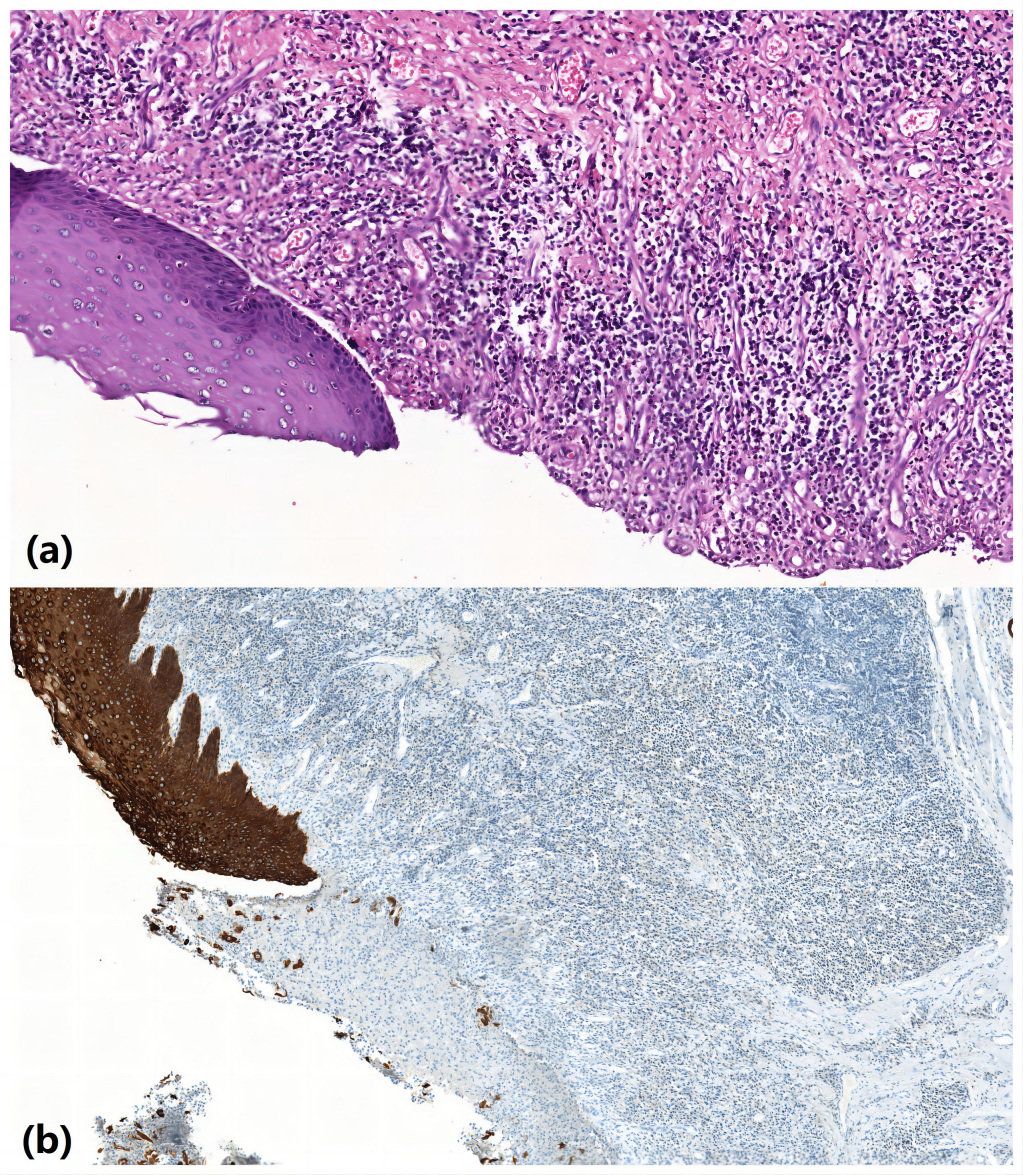
Pathological esophagus pathology revealed separation of squamous epithelium separated from submucosal tissue in some areas of esophageal mucosa, ulcer formation, granulation tissue hyperplasia, and some glands with squamous metaplasia. **(A)** HE ×200; **(B)** CK immunocytochemistry ×200.

Postoperative pathological esophagus pathology revealed separation of squamous epithelium separated from submucosal tissue in some areas of esophageal mucosa, ulcer formation, granulation tissue hyperplasia, and some glands with squamous metaplasia. These results were consistent with the mucosal changes of RDEB patients (**Figures 4A, B**). Gastroscopy was planned to be performed 5 months after surgery, but the patient refused the examination for fear of mucosal trauma that might occur during gastroscopy operation. However, the patient was given esophageal barium swallow examination (**Figure 3B**). The patient complained of obstruction when swallowing large pieces food 29 months after the operation, and was given esophageal barium swallowing test (**Figure 3C**). On 31 months after surgery, the patient underwent gastroscopy (**Figure 3D**). By the time this article was submitted, the patient had been followed up for 42 months. There was a sense of obstruction when swallowing large pieces food, accompanied by mild food residue in the pharynx and gastroesophageal reflux symptoms, and the body weight increased by about 12 kg.

## 3 Discussion

DEB, as a type of EB, is caused by the low expression of type VII collagen due to *COL7A1* mutation, which results in the abnormal connection fiber between the epidermis and dermis (8). It is mainly characterized by scar-prone skin and mucosa. Digestive tract and respiratory tract are often involved, and the esophagus is the most commonly involved site, besides the skin (9). The incidence of comorbid RDEB and esophageal stenosis can reach 80% (6).

There is no effective clinical treatment for EB. At present, research mainly focuses on the treatment of primary genetic abnormality and secondary inflammation of EB. Among the treatments currently attempted include gene therapy based on gene replacement and gene editing, cell therapy based on fibroblast and bone marrow transplantation, recombinant protein therapy, and small molecule and drug reuse therapy (10). *In vitro* gene replacement based on full-length *COL7A1* cDNA transduction by efficient retrovirus has been used to treat the restore function of endogenous genes (11). By establishing an inactivated lentivirus platform and intradermal injection of COL7A1-modified autologous fibroblasts, the expression of fibroblasts in the skin of EB patients can be improved and increased collagen VII levels to achieve the therapeutic goal (12). Before some scholars, in a different approach, have used losartan to improve the clinical symptoms of recessive dystrophic epidermolysis bullosa by inhibiting the activity of TGF-β and reducing the inflammatory process mediated by TGF-β signaling pathway, thus slowing the formation of scar (13).

Dysphagia can be relieved through endoscopic esophageal dilation surgery or esophageal stent implantation, but there is no effective strategy to control further development of esophageal inflammation. Therefore, a large number of patients experience esophageal stenosis recurrence. For patients with severe esophageal stenosis or near-total atresia, gastrostomy surgery is often used to achieve the body’s nutritional intake needs (7). The jejunum transplantation approach was used to repair severe esophageal stenosis. Compared with endoscopic dilation surgery, although the trauma is greater, it can directly remove the diseased esophagus, reduce the pain of patients undergoing repeated esophageal dilation, and reduce the probability of recurrence. Compared with gastrostomy, it can restore patients’ oral feeding function and greatly improve patients’ quality of life. Through a 42-month post-surgery follow-up observation, we believe that it is an effective method for treatment of EB complicated with severe esophageal stenosis.

The preoperative esophageal barium meal examination of the patient suggested that the length of the severe stenosis was 4 cm, but the intraoperative exploration revealed that the actual length of the stenosis was about 6cm. However, the esophageal tissue with a length of about 8 cm was finally removed because we found that the mucosa had clearly visible ulcerative changes in the esophageal lumen without stenosis. This was consistent with the mucosal range suggested by the preoperative enhanced CT examination of the neck.

For jejunum transplantation, jejunal tissue 100 cm away from the Qu’s ligament was selected for transplantation. During transplantation, it was found that the diameter of the jejunal tissue was larger than that of normal esophagus. Some reports suggest choosing intestinal tissue 30-40 cm away from Qu’s ligament because the diameter of the intestinal tract here is similar to that of esophageal canal (14). However, we believe that the selection of jejunum with larger diameter can fully expand the inner diameter of the lumen, prevent the occurrence of anastomotic stenosis, and reduce the incidence of jejunal peristaltic dysfunction when performing end-to-end anastomosis with normal esophagus. Before transplantation, we considered that the direction of spontaneous peristalsis of the jejunum should be consistent with the order of swallowing food to reduce the risk of gastroesophageal reflux. In the wound healing period, acid suppression therapy was applied to avoid the occurrence of anastomotic fistula and anastomotic stenosis caused by gastroesophageal reflux. During anastomosis, we inserted a gastric tube into the stomach through the nose. After the operation, gastrointestinal decompression was given to prevent acid reflux. Meanwhile, enteral nutrition support was also provided in the early stage to promote the healing of the surgical wound. Although Yoo et al. reported an increased risk of esophageal stenosis when nasogastric tube is placed for a long time (15). In this case, it was believed that a nasogastric tube kept for 2 weeks would not increase the risk of postoperative esophageal stenosis in the patient. However, a better approach would have been to use a combination of nasogastric tube decompression and nasoenteric tube nutritional support.

After jejunum dissociation, literature suggested the lavage of intestinal cavity and mesenteric vessels (16) to reduce thrombosis and improve the success of jejunum transplantation. However, because of the possibility of vascular wall damage due to DEB-induced congenital skin and mucous membrane lesions, lavage was not used. Therefore, after dissociation, the jejunum was only soaked in warm saline, and no intestinal cavity or vascular lavage was used. Postoperative complications such as abnormal intestinal blood flow and necrosis did not occur.

Traditional Chinese medicine acupuncture and umbilical moxibustion were administered to accelerate the recovery of intestinal peristalsis after operation. In addition, assessment of the blood flow in the transplanted jejunal anastomotic vessels using bedside ultrasound scanning (17), revealed normal postoperative blood flow. In case of blood flow obstruction of anastomotic vessels, immediate surgical exploration is recommended, followed by anastomosis of re-selected transplanted vessels, if necessary, to improve the survival rate of the transplanted jejunum. The symptom of bloody stools after nasal feeding was more likely due to the intestinal dysfunction and intestinal nutrient solution intolerance of the patient. However, intestinal mucosal lesions caused by DEB could not be excluded. Through our conservative treatment, the patient’s hematochezia symptoms gradually disappeared. In addition to the above related complications, anastomotic infection, graft necrosis, dietary cough, aspiration pneumonia, postoperative severe esophageal stenosis and other possible complications had been fully prepared in the preoperative discussion. If serious complications occur, we may take a second operation, strengthen the intraoperative cavity dressing change, give adequate anti-infection therapy and other ways to manage.

After 42 months of follow-up, there was a certain degree of stricture in the pharyngian-jejunal anastomosis, which was considered to be caused by postoperative scar, but this stenosis did not lead to serious dysphagia. Postoperative cicatricial stricture was expected, so we tried to enlarge the anastomosis during the operation, and indwelling gastric tube was placed after the operation to prevent acid reflux. Endoscopic dilation and esophageal stent implantation may be used if severe anastomotic stenosis occurs after esophageal surgery. Regarding the timing of surgery, the stable stage of the patient’s clinical symptoms should be chosen, rather than the progressive stage. Gastroscopy revealed no mucosal ulcers in the esophagus and the transplanted jejunum. We used free jejunum to reconstruct the upper digestive tract of a patient with RDEB complicated with severe esophageal stenosis. This successful operation provides a new and effective treatment for patients with RDEB to improve clinical symptoms and their quality of life.

## Data availability statement

The original contributions presented in the study are included in the article/supplementary material. Further inquiries can be directed to the corresponding author.

## Ethics statement

Ethical review and approval was not required for the study on human participants in accordance with the local legislation and institutional requirements. The patients/participants provided their written informed consent to participate in this study. Written informed consent was obtained from the individual(s) for the publication of any potentially identifiable images or data included in this article.

## Author contributions

ZX and YCH contributed to conception and design of the study. YZ and XX organized the database. ZX and YJH completed the manuscript. BY and TH were in charge of literature retrieval. All authors contributed to the article and approved the submitted version.
